# Decoupling Environment-Dependent and Independent Genetic Robustness across Bacterial Species

**DOI:** 10.1371/journal.pcbi.1000690

**Published:** 2010-02-26

**Authors:** Shiri Freilich, Anat Kreimer, Elhanan Borenstein, Uri Gophna, Roded Sharan, Eytan Ruppin

**Affiliations:** 1 The Blavatnik School of Computer Sciences, Faculty of Life Sciences, Ramat Aviv, Israel; 2 Sackler School of Medicine, Faculty of Life Sciences, Ramat Aviv, Israel; 3 School of Mathematical Science, Faculty of Life Sciences, Ramat Aviv, Israel; 4 Department of Molecular Microbiology and Biotechnology, Faculty of Life Sciences, Ramat Aviv, Israel; 5 Department of Biomedical Informatics, Columbia University, New York, New York, United States of America; 6 Department of Biological Sciences, Stanford University, Stanford, California, United States of America; 7 Santa Fe Institute, Santa Fe, New Mexico, United States of America; University of Washington, United States of America

## Abstract

The evolutionary origins of genetic robustness are still under debate: it may arise as a consequence of requirements imposed by varying environmental conditions, due to intrinsic factors such as metabolic requirements, or directly due to an adaptive selection in favor of genes that allow a species to endure genetic perturbations. Stratifying the individual effects of each origin requires one to study the pertaining evolutionary forces across many species under diverse conditions. Here we conduct the first large-scale computational study charting the level of robustness of metabolic networks of hundreds of bacterial species across many simulated growth environments. We provide evidence that variations among species in their level of robustness reflect ecological adaptations. We decouple metabolic robustness into two components and quantify the extents of each: the first, environmental-dependent, is responsible for at least 20% of the non-essential reactions and its extent is associated with the species' lifestyle (specialized/generalist); the second, environmental-independent, is associated (correlation = ∼0.6) with the intrinsic metabolic capacities of a species—higher robustness is observed in fast growers or in organisms with an extensive production of secondary metabolites. Finally, we identify reactions that are uniquely susceptible to perturbations in human pathogens, potentially serving as novel drug-targets.

## Author Summary

When a species is grown under optimal conditions the single-knockout of most of its genes is not likely to affect its viability. The resilience of biological systems to mutations is termed genetic robustness and its extent across different species has not yet been systematically described. Since the deletion of a gene can have varying consequences depending on the environmental conditions, the extent of species' genetic robustness reflects both the range of conditions (or environments) in which it can survive as well as the availability of alternative cellular routes (compensating for a gene's loss of function). Here, we developed a computational model for estimating the essentiality of metabolic reactions across natural-like environments and applied it to chart species' level of genetic robustness, providing the first systematic description of genetic robustness across species. Studying robustness across a wide collection of natural-like environments enables one to stratify, for each species individually, the extent of environmental-dependant and independent robustness and hence advances our understanding of its evolutionary origins. Our main finding is that the level of environmental dependent robustness is associated with the lifestyle of a species (i.e., specialists versus generalist), whereas the level of environmental-independent robustness is associated with its metabolic production capacities.

## Introduction

Systematic deletion studies have shown that under laboratory conditions the large majority of genes in the genome are dispensable, and that in many cases dispensability depends on the experimental setting [1]. These studies have reinforced the notion of robustness of biological systems, which denotes the invariance of phenotypes in the face of perturbations [2]. Two types of perturbations are encountered by biological systems: genetic alterations and environmental variations. Genetic robustness of a biological system is viewed as its ability to continue functioning following mutations [3],[4], while environmental robustness refers to buffering against external changes (e.g., changes in temperature and salinity, or in the availability of nutrients). Genetic robustness can be studied at various levels of biological organization, from the molecular level to the organism level, where a state is considered to be robust if a mutation has an insignificant effect on the trait examined [2].

Environmental robustness differs greatly across bacterial species: whereas some species exhibit an impressive ability to proliferate in a wide spectrum of habitats, others demonstrate highly specialized nutritional requirements. In free-living organisms, new pathways have evolved by acquiring reactions that put external nutrients into metabolic use allowing species a greater choice in their metabolic requests [5]. The genetic implications of the selective pressure to increase the nutritional repertoire make environmental robustness and genetic robustness intertwined. The contribution of environmental robustness to genetic robustness is demonstrated by the following example: the evolution of a new metabolic pathway allows a species to put a new external metabolite (metabolite A) into metabolic use as an alternative to an existing pathway making use of metabolite B, hence promoting environmental robustness. Under such conditions where both A and B are available (nutrient-rich conditions), the species will also gain genetic robustness in front of mutations in one of the corresponding pathways (in case of a mutation preventing the use of metabolite B the species can use the alternative pathway utilizing A and vice versa). The effect of environmental robustness on genetic robustness in metabolic networks was studied in detail in yeast [6],[7]: more than half of the genes that were nonessential for growth under nutrient-rich conditions were found to be active under eight restricting growth conditions [7]. However, as environmental robustness cannot fully explain the dispensability of genes, two other hypotheses have been proposed for the evolution of genetic robustness [2]: First, an *adaptive* origin – i.e., a direct, natural selection in favor of genes which allow a species to endure genetic perturbations (initially suggested by Fisher to explain the observed dominance of wild-type alleles to the overwhelming majority of deleterious mutations [8]). Second, an *intrinsic* origin– i.e., genetic robustness has evolved as a byproduct of natural selection in favor of other, adaptive, traits. In the context of dominance, selection for increased metabolic flux is a widely accepted explanation for the recessive nature of most mutations [9]–[11]. Since metabolic enzymes act as part of large, multi-enzymes, networks, single-loci mutations (resulting in 50% activity of the corresponding enzyme in the heterozygote) are not expected to affect the overall behavior of the system. Hence such mutations are not detectable at the phenotypic level and mutants are considered to be recessive [12]. For genetic robustness, one can similarly argue that robustness is intrinsic to the optimization of some phenotypes, and has evolved as a byproduct of a selective pressure for increasing steady-state metabolic fluxes via the incorporation of alternative metabolic pathways [2],[13],[14]. Notably, there is an essential difference between man-made systems, where robustness is built in on purpose, and biological systems that are shaped by natural selection at the population level, in which non robust systems can survive if reproduction rate compensates failure rate.

Few recently developed methods allow one to systematically address the influence of the environment on metabolic network structure. The network expansion method – a method for generating the set of all possible metabolites that can be produced from a set of compounds - permits the reconstruction of networks in different metabolic environments [15]. Using the network expansion approach, generic sub-networks (describing a collection of metabolic reactions across known genomes rather than the reactions tenable within any specific organism), expanded under different collection of metabolites, were shown to be highly robust against the elimination of mutations [16],[17]. The network expansion method has been helpful in uncovering the role played by some specific metabolites on the evolution of metabolic networks. The introduction of oxygen to the atmosphere, for example, was demonstrated to be coupled with the appearance of many new pathways and reactions, considerably increasing the complexity of the metabolic networks of aerobic species beyond that reachable by any anoxic network [18]. Studying the species-specific expansion of networks given various metabolite combinations has permitted the grouping together of organisms with similar metabolic capabilities [19]. Following the introduction of the expansion method, other related topological approaches have been developed to provide predictions for the network-specific set of externally consumed metabolites, as inferred by the structure of the corresponding metabolic network [20],[21]. These methods aim to define the minimal set of externally acquired compounds – i.e., these metabolites that cannot be synthesized from other compounds and permit the production of all other compounds in the network. Here, we combined the expansion algorithm and an algorithm for predicting species-specific metabolic environments (The seed algorithm [20]). By integrating these two algorithms, we provide a new model that not only predicts robustness across species (as was previously done using the expansion method [17]), but also examines robustness across environments and hence provides predictions for condition-dependent and independent reactions. Taking this integrative approach, we aim to characterize the level of species-specific robustness and, for each species individually, to decouple between robustness which is condition-dependent (due to reactions which are essential in some environments but not in other) and robustness which is condition-independent. We start by describing our model and testing its biological plausibility. We then use it to systematically characterize the level of robustness across bacterial species (condition dependent and independent). Finally, we apply the model for studying whether dispensability is a directly selected feature.

## Results

**Figure 1:**
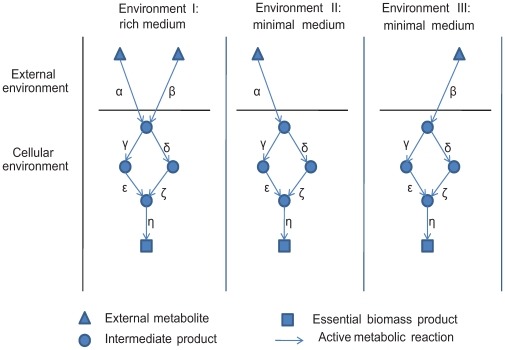
Illustration of a simple metabolic network producing an essential constituent of biomass on different growth media.

The buffering capacity of metabolic networks was previously predicted using *in silico* metabolic flux analysis approaches [22]–[32] but the underlying stoichiometric metabolic models (providing the quantitative relationships between the reactants and the products of each reaction) are available for only a few selected species. Topology-driven approaches (requiring only the network topological backbone and not a full blown stoichiometric model), although providing only qualitative predictions for the activity of a reaction (active/not active) rather than a quantitative estimate of the fluxes it carries, have been previously shown to predict the *in vivo* essentiality of genes with considerable accuracy [33],[34]. Such methods unravel *topological*, *network genetic robustness* (NGR), which refers to the network's ability to buffer mutations via the existence of alternative pathways, and is distinct from robustness that arises from the presence of alternative genes (duplicates or functional analogs) [4],[16],[29],[30]. Here, we studied species-specific topological robustness by applying a topology-driven computational approach for predicting an organism's viability in a given environment, estimated according to its ability to produce a set of essential biomass metabolites (Figure 1). Taking a similar approach to [21], these metabolites were chosen because they are deemed essential for life in all known bacteria and include amino acids, nucleotides and essential co-factors (Methods).

We quantify metabolic NGR in 487 bacterial organisms. For each species we simulate its growth on a rich medium and systematically delete each reaction in turn. Under each deletion, we test the ability of the metabolic network to produce key biomass metabolites (Methods). A specific rich medium was individually computed for each species by employing a previously developed ‘reverse ecology’ algorithm [20] that computes the full set of metabolites that an organism extracts from its environment (Methods). Growth simulation was done by using the expansion method [15] – an approach where networks of increasing size are constructed starting from an initial set of substrates (the seed) by stepwise addition of those reactions whose substrates are produced in the current core network (i.e., compounds present in the seed or provided as product by reactions incorporated in earlier steps). Here, the expansion method was used to construct the species-specific metabolic networks following each deletion, given the species-specific rich environment. In the toy example illustrated in Figure 1 this procedure leads to the identification of the two external metabolites, based on the topology of the metabolic network. NGR is calculated as the fraction of non-essential reactions (i.e., those reactions whose absence is compensated by the presence of alternative routes) out of all network reactions. In Figure 1, η is the only essential reaction out of 7 reactions and the NGR of the network is 6/7. The topological-based essentiality predictions show good agreement in two species where essentiality data has been assembled via experimental knock-out studies – *Escherichia coli* and *Bacillus subtilis* – yielding an accuracy of 0.86 and 0.85 respectively (Methods). Taken together, the computation of species-specific rich growth media results in an ensemble of 487 environments, representing a sample of the ecological niches that different bacterial species can inhabit.

To elucidate the contribution of *environmental* robustness to genetic robustness across species and lifestyles, we first assess the viability of all 487 species across these 487 sample environments (in Figure 1, for example, we identify two viable environments – environments II and III). We consider the fraction of the environments in which a given species is viable as a measure of its *environmental robustnesss* [35]. This measure significantly correlates with two (general, non-metabolic) established measures of variability of species' habitats (Fraction of regulatory genes: 0.44, *P* = 9e-8 [36]; Environmental complexity: 0.33, *P* = 3e-4 [37], Spearman correlations; Methods), showing that as a general trend (though not in all cases) high environmental robustness in observed in generalist species. As expected (according to [38]), the environmental robustness of species also significantly correlates with the modularity of their metabolic networks (−0.43, *P*<2e-16, Methods).

**Figure 2:**
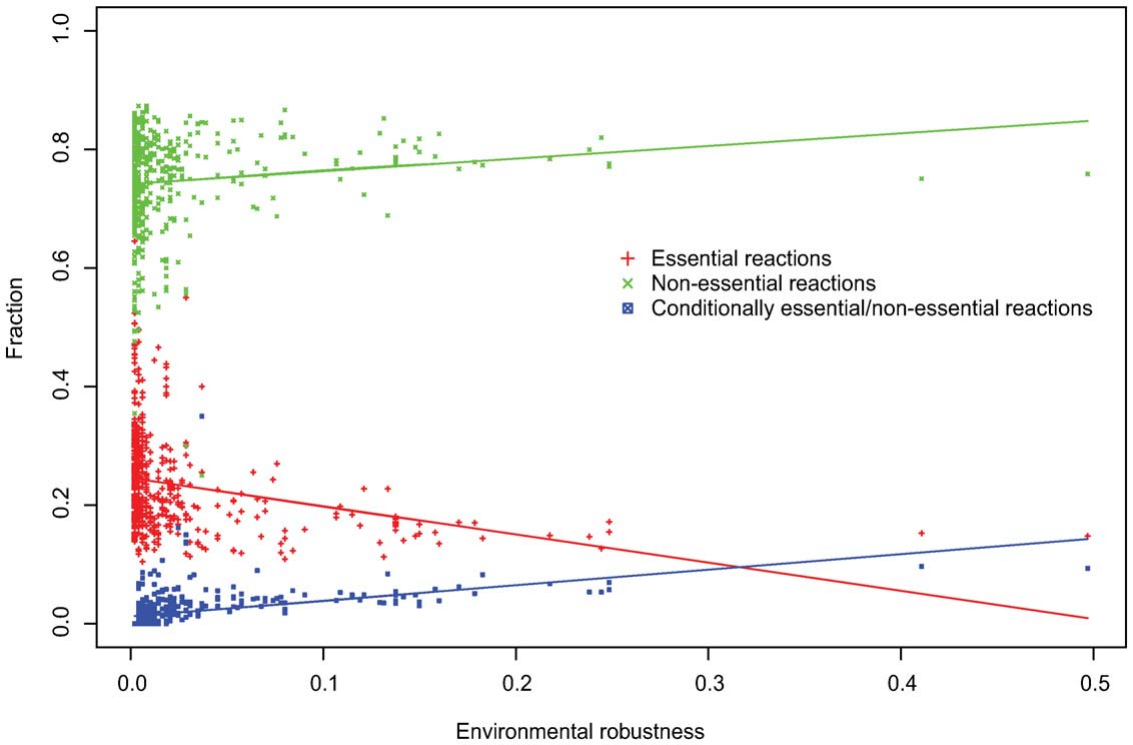
The distribution of non-essential, essential and conditionally-essential/non-essential reactions versus environmental diversity across the 487 organisms studied. Lines represent the linear regression calculated for each group.

Following using the collection of 487 environments (constructed by calculating the optimal metabolic environment of each species in the analysis and hence representing a sample of the ecological niches that different bacterial species can inhabit, Methods) for predicting species-specific environmental robustness, we use it for the identification of conditionally essential reactions. In every viable environment of a given organism (that is, a collection of metabolites that allows the production of all target metabolites of a given species) we systematically delete all its metabolic reactions (single reaction at a time). This leads to the identification of *conditionally essential/non-essential reactions* – reactions that are essential in some viable growth environment of the organism but are non-essential under other, more favorable, conditions such as its own species-specific rich medium (reactions α and β in Figure 1; Methods). The resulting matrix, describing the essentiality of all metabolic reactions across the 487 by 487 species and environments, is given in Text S1 note 1. The metabolic reactions of each species can hence be divided into three non-overlapping groups: (i) *(unconditionally) essential reactions* – i.e., reactions that are essential over all growth media (reaction η in Figure 1); (ii) *conditionally essential/non-essential reactions* (reactions α and β in Figure 1); and (iii) *(unconditionally) non-essential* reactions – that are backed-up over all growth media (reactions γ, δ, ε and ζ in Figure 1, and see Text S1 note 2 for evidence that we recover the large majority of conditionally essential genes). For each network we compute a *condition-independent NGR score (ciNGR)* that denotes the fraction of the non-essential reactions (group iii) out of all reactions and a *condition-dependent NGR score (cdNGR)* which denotes the fraction of the non-essential and conditionally-essential/non-essential reactions (group iii and group ii) out of all reactions. The fraction of each reaction's group (non-essential, essential, conditionally-essential/non-essential) over all species is shown in Figure 2. In *Escherichia coli*, where 83% of the genes were experimentally shown to be dispensable under aerobic growth in rich medium [39],[40], we observe a corresponding fraction of 0.78 non-essential reactions (Table S1). In host-dependent species the observed fraction of non-essential reactions (ciNGR) is typically markedly lower (0.35 in *Mycoplasma genitalium* for example), in accordance with the experimentally observed range of 20% to 60% dispensable genes [39]. Overall, in most species we observe a large fraction of non-essential reactions closely scattered around a mean of 0.75 (Table S1, Figure 2).

**Figure 3:**
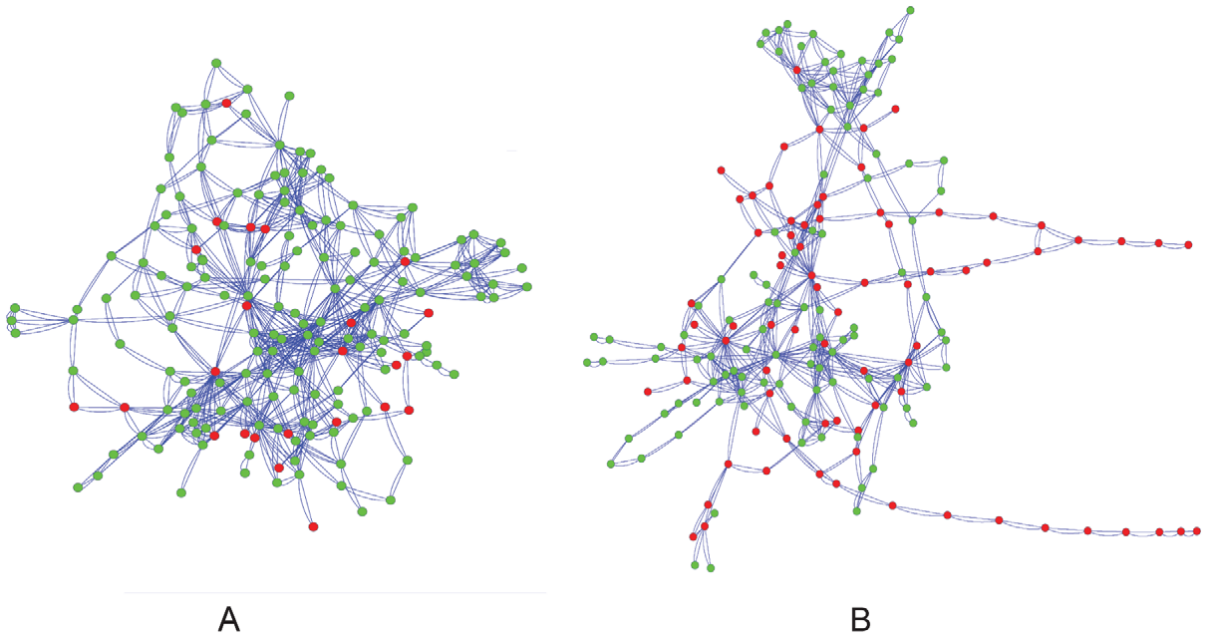
The metabolic networks of species with similar network size and different topological properties. (A) *Clostridium botulinum*: Network size, 189; connectivity, 5.2; centrality (mean shortest path), 3.7; robustness (NGR), 0.85. (B) *Helicobacter acinonychis*: Network size, 191; connectivity, 4.1; centrality (mean shortest path), 5.4; robustness (NGR), 0.56. Red circles - essential reactions; green circles - non-essential reactions.

We further studied how the topology of the metabolic networks relates to their level of observed robustness. For this, we looked at two topological characteristics measured for each network: network connectivity – describing the average number of neighbors each protein-node has, and network centrality – describing the average centrality of its node members where the centrality of each individual node is determined by calculating the mean shortest path between the node and all other nodes in the network (Methods). The overall robustness of the network (cdNGR) is positively correlated with its topological properties including network size, network connectivity and network centrality (size of network: 0.61, *P* = 0; connectivity: 0.77, *P* = 0; mean shortest path: −0.65, *P* = 0; Spearman correlations; Methods). This shows that an array of topological properties is related to network robustness and that the structure of the network, and not only its size, has functional significance. The associations between condition-dependent NGR, centrality and connectivity remain significant when controlling for the effect of network size (Text S1 note 3), providing a system-level support to the view that network connectivity contributes towards a better compensation for loss-of-function mutations [41],[42]. The association between topological properties, robustness, and species' lifestyle is visualized in Figure 3 where we show two metabolic networks of similar size (∼200 reactions) that greatly differ in both their topological properties and their robustness. The metabolic network of the free-living *Clostridium botulinum* is more central and connected than the network of the host-associated *Helicobacter acinonychis*, and correspondingly exhibits a remarkably higher robustness.

Next we quantified the robustness of each reaction in each species as the fraction of its non-essential entries across all viable environments, marking its degree of conditional-essentiality in each species (from 0 – essential across all environments, to 1 – non-essential across all environments). For each species, we studied the association between the species-specific level of reaction's essentiality and other topological and evolutionary characteristics. In accordance with experimental data from yeast [41], non-essential reactions tend to be more central and highly connected than essential reactions (Methods; Text S1 Note 4). Hence, our data provide a large-scale, cross-species support to findings observed at the single-species level. The robustness of reactions is also associated with evolutionary conservation: in the large majority of species (83%) we find a significant positive correlation (P<0.05) between a reaction's robustness and its conservation, as inferred from the phylogenetic distribution of reactions across species (mean correlation – 0.21, maximal correlation – 0.37; Text S1 Figure 2). At the species level, this correlation is absent primarily in host-associated species, where the robustness of reactions is likely to be affected by the host-microbe interactions (Text S1 Figure 2). Notably, when considering the robustness of reactions across all species and environments (i.e., a reaction-specific score describing its mean level of essentiality across all species) the correlations between essentiality and conservation is markedly higher than that observed when considering each species alone (0.58 P<2.2e-16, Spearman, and compare with Text S1 Figure 2) further testifying to the utility of the large scale investigation performed here.

Examining the distribution of the reaction categories within major metabolic classes reveals two metabolic categories that are highly enriched in non-essential reactions: nucleotide metabolism and carbohydrate metabolism (*P* = 0, Fisher test) (Text S1 note 5). Conversely, reactions functioning in amino-acid biosynthesis contain significantly more essential reactions (conditionally and un-conditionally) than expected by chance (*P* = 0, Fisher test), hence, due to the over-representation of essential reactions, comprising a particularly non-robust to mutations functional category. The level of essentiality of reactions associated with two very basic metabolites, oxygen and ATP, is an interesting case of marked dissociation. We find that oxygen-utilizing reactions are highly backed-up (fraction of non-essential appearances in oxygen-utilizing reactions versus all reactions: 0.96 and 0.88 respectively; *P* value 5e-5 in a Wilcoxon test; Text S1 Note 6 and 7, Table S4). There are several possible explanations for the high level of redundancy of oxygen utilizing reactions: First oxygen was introduced into the atmosphere after the appearance of cellular life forms, where oxygen-dependant reactions were shown to augment more ancient, pre-oxygen reactions [18]. Second, oxygen is a limiting factor across many environments and hence the availability of alternative pathways allows species to alternate between pathways, according to the environmental conditions at a given time. Notably, reactions utilizing other redox molecules such as NAD also show high level of redundancy (fraction of backed appearances in NAD-utilizing reactions: 0.93; *P* value 5e-3 in a Wilcoxon test; Text S1 Note 6), possibly due to the occurrence of alternative pathways using NADP or other electron acceptors. However, oxygen-utilizing reactions are still significantly more backed-up (i.e., non-essential) than NAD utilizing reactions (*P* value 0.017 in a Wilcoxon test; Text S1 Note 6). ATP-dependent reactions, on the other hand, have significantly low levels of robustness (fraction of backed appearances in ATP-utilizing reactions: 0.79; *P* value 1e-4 in a Wilcoxon test; Text S1 Note 6). The least robust (most essential) reactions across bacterial species are those performed by ATP-dependent amino-acyl tRNA synthethases (Table S2), a class of highly conserved enzymes [43] that are known to be essential across species [44]. One potential explanation for the high level of essentiality of ATP-dependent reactions may be that such reactions, being thermodynamically unfavorable in the forward direction when not coupled to ATP hydrolysis, are not likely to have other, spontaneous, alternatives.

Figure 2 displays the correlation between the species distribution of the reaction types (non-essential, essential and conditionally essential/non-essential) with environmental robustness. Notably, the fraction of ciNGR (unconditionally *non-essential* reactions - green dots in Figure 2) is not strongly affected by the species' level of environmental robustness (the number of environments in which it is viable). In contrast, the fraction of conditionally-essential/non-essential reactions exhibits a remarkably high correlation with environmental robustness (0.81, P<2e-16, Spearman), which remains significant when controlling for the effect of network size (Text S1 Note 8). A strong correlation between the fraction of conditionally-essential/non-essential reactions and environmental robustness is also observed when using an alternative set of 20,000 environments constructed by random shuffling of the seeds from the original environments while maintaining their original distribution (0.76, P<2e-16, Spearman; Text S1 Note 2). Our findings thus provide direct large-scale evidence that genetic robustness is associated with environmental robustness. Although such association is expected considering both the nature of the model as well as the biological setting it aims to recapture, the use of the approach for the characterization of condition-essential/non-essential reactions demonstrates that environmental diversity by itself cannot fully account for the level of robustness observed, whereas conditional-independent robustness is observed across all species examined including the most specialized and most diverse ones (Figure 2). Notably, the fraction of conditionally-essential/non-essential reactions almost invariably does not exceed 20% of the total metabolic reactions in the dataset, in line with previous experimental findings indicating that niche-specificity by itself cannot explain the dispensability of a significant fraction of the genes [6],[7],[45]. While these results provide a fair estimate of the contribution of nutritional factors, other environmental factors (e.g., temperature, salinity, etc.) that go beyond the current model probably lead to an overall larger environmental contribution.

**Figure 4:**
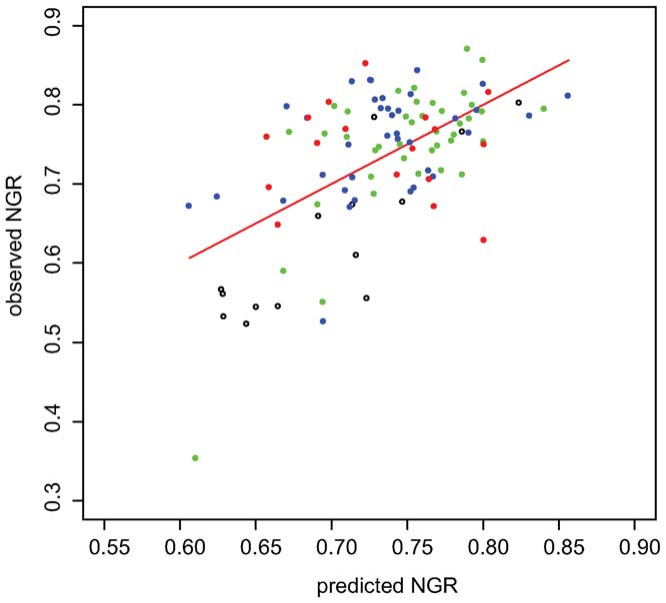
Observed versus predicted NGR. Predicted values are derived from a generalized linear predicting NGR from the growth rate and fraction of secondary metabolites of each species. Growth rate data was available for 109 species including 17 anaerobic (red), 37 aerobic (blue), 40 facultative (green), 4 microaerophilic, and 11 unknown.

As environmental diversity mainly affect the robustness of condition-dependent reactions and not condition-independent reactions (Figure 2), we turn to study the level of association between metabolic activity and the level of robustness of the latter group (ciNGR), aiming at revealing its pattern of association with other phenotypic characteristics. Notably, no correlation is observed between the fraction of ciNGR and the fraction of conditional-essential/non-essential reactions, further supporting the view that the evolution of the condition-independent component of genetic robustness is derived by different selective forces than the condition-dependent component. We use growth rate data to account for the growth capacity of an organism and additionally measure the fraction of metabolic reactions dedicated to the production of its secondary metabolites (Methods) [46]. A generalized linear model based on both measures is employed to predict network robustness values and yields a fairly marked correlation with the observed NGR scores (0.59 Pearson, P = 1e-11, Figure 4), providing a significantly improved fit over the results obtained while using each measure individually (Text S1 Note 9). This association, though by itself cannot infer causality, provides support to the notion that a need to increase metabolic capacities has been a driving force in the evolution of genetic robustness in bacterial metabolic networks [2]. Unlike the correlation between metabolic activity and condition-independent robustness reported above, the correlation between metabolic activity and condition-dependent robustness is insignificant when controlling for the effect of other co-associated factors (Text S1 Note 10). Interestingly, the predictor provides a good approximation for the level of genetic robustness in facultative bacteria and a more moderate approximation in aerobic bacteria (facultative: 0.70 P 5e-7; aerobic: 0.43 P 0.007; Pearson correlations), but an insignificant one for anaerobic bacteria (Figure 4). Indeed, it has been previously suggested that oxygen-tolerant bacteria (facultative and aerobic) have developed an array of alternative metabolic pathways of different energetic costs, whose activity they delicately balance to optimize their metabolic yield given the environmental conditions [47],[48]. Notably, information on the rate of growth is only available to less than fourth of the species examined, and many pathways involved in secondary metabolism are yet to be revealed. Hence our estimate of metabolic capacity only reflects current state of knowledge. The availability of growth rate information for additional species, as well as the characterization of additional secondary pathways will allow more accurate predictions of species' metabolic capacities.

To study whether genetic robustness has evolved directly in an adaptive manner we use an approach suggested by [7] and examine the association between network-level robustness (the existence of alternative pathways) and gene-level robustness (the existence of a duplicate gene or of a functional analogs) [2],[42],[45] across all species studied. If the network dispensability of reactions would have adaptively evolved to provide resilience to mutations, one would expect that duplicate genes of non-essential reactions would be preferentially lost while duplicate gene copies of essential genes would be preferentially maintained, as they provide gene-level back-up to essential reactions. Yet, in accordance with previous findings in yeast [7], we find that essential reactions are not more enriched in multi-copy genes (Text S1 Note 11). We further looked the level of genetic robustness at *Rubrobacter xylanophilus* and *Deinococcus radiodurans* - extermophyls which are exposed to high levels of radiation (Text S1 Notes 1). The NGR values of both species do not significantly differ from those observed in other bacteria. (0.74 and 0.77 respectively, compared with a mean value of 0.75 over all species), and hence do not support an association between high level of genetic robustness and high rate of genetic perturbations, as can be expected in case of adaptive origin of robustness.

**Figure 5:**
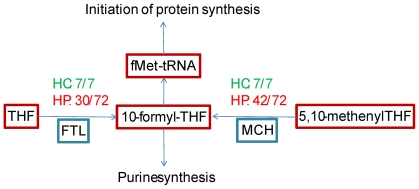
Distribution of pathways for the synthesis of 10-formyltetrahydrofolate in human pathogens and human commensal organisms. Maroon squares: metabolites; blue squares: reactions. MCH: methenyltetrahydrofolate cyclohydrolase; FTL: formyltetrahydrofolate synthetase; HC: human commensals; HP: human pathogens. 10-Formyltetrahydrofolate acts as a formyl donor in purine biosynthesis, and for formylation of methionyl-tRNA required for producing fMet-tRNA – a molecule required in most bacterial species for initiating protein synthesis. All human commensals (7/7) contains two alternative routes for the production of 10-formyltetrahydrofolate. Only 28 out of 73 human pathogens which have MCH contain the alternative route, making MCH essential in the remaining 45 organisms. These 45 pathogenic organisms include several *Shigella*, *Salmonella* and *Mycobacterium* species (the full list of species and the essentiality of MCH and FTL is provided in Text S1 Note 13 and in Table S5). The approach presented here can easily be generalized for highlighting essentiality in other groups of medical, ecological or agricultural interest.

Beyond evolutionary insights about the extent and origins of metabolic robustness, our approach permits the high-throughput identification of essential reactions across species and can be applied for delineating species-specific (or group-specific) essentiality. Potential antibiotic drug targets, for example, can be identified by revealing reactions that are backed up in commensal human bacteria while essential in human pathogens. Methenyltetrahydrofolate cyclohydrolase (EC 3.5.4.9) is an example for a widely distributed reaction (present in most species) that is non-essential across all non-pathogenic human bacteria in our data, while essential in many human pathogens (Figure 5). In many pathogenic species methenyltetrahydrofolate cyclohydrolase catalyses the only reaction for the production of 10-formyltetrahydrofolate, an essential metabolite for the translation process in bacteria [49],[50]. In human commensal organisms, an alternative route for the production of 10-formyltetrahydrofolate is available via a reaction catalyzed by Formyltetrahydrofolate synthetase (6.3.4.3). Notably, methenyltetrahydrofolate cyclohydrolase has known inhibitors, which have little activity in mammalian cells and are therefore selective [50]. Additional examples of pathogenic-specific essential reactions are provided in Text S1 Note 13 and in Table S6.

## Discussion

Overall, our analysis charts out the robustness of the metabolic system across a wide variety of bacterial species and growth media. Several limitation of this analysis should yet be acknowledged. The predictions for the essentiality of reactions, as well as the set of growth environments, are based on a topological network-based computation. Hence, this analysis ignores many other properties of metabolic reactions such as stochiometry, rate, and dynamics. Incorporating these properties into the metabolic network model can potentially yield more accurate results [51]. Nevertheless, metabolic network topologies can readily be obtained for hundreds of species, allowing a phylogenetic, large-scale analysis [37] and may thus delineate emerging patterns in the metabolic data. This broad perspective enables the elucidation of general principles underlying the structure and evolution of metabolic networks [16],[17],[52]. Finally, our predictions of reactions' essentiality are shown to be in general agreement with experimental observations, in the few cases where the latter exist.

Several recent studies have used topological-based approaches for systematically revealing the structural properties of metabolic networks. The expansion algorithm was applied for estimating the level of robustness in reference sub-networks, comprising all reactions present in the KEGG database (irrespective of the organism in which they have been found) [16], as well as for conducting a comparative study of the level of robustness in species-specific sub-networks produced by the expansion algorithm under a given combination of external resources [17]. These studies clearly testify for the high robustness of metabolic sub-networks, where the sensitivity following deletion of reactions decreases as the size of the sub-network increases [17]. Here, we introduce a computational approach that integrates together algorithms which were previously used individually for studying the structure and evolution of metabolic network: The expansion algorithm was used for studying robustness under different environments and in different species (though in a given environment); The seed algorithms were used to predict the nutritional environment of species [20],[21]. By integrating these two algorithms, we provide a new model that provides predictions for condition-dependent and independent robustness across many species and environments. By conducting a large-scale comparative study we provide evidence that variations among species in their level of network genetic robustness (condition-dependent and independent) reflect adaptations to different ecological niches and lifestyles. Notably, beyond robustness, other features of metabolic networks such as the collection of enzymatic functions [53] and the ability to utilize external nutrients [19] also reflect environmental adaptations, where in many cases the metabolic capacities of species are better associated with their lifestyle than with their phylogeny. Alternative lifestyle characteristics are associated with the two types of robustness: the extent of conditional-dependent robustness is strongly associated with the environmental diversity of species (specialized or generalist), and the extent of condition-independent robustness is associated with the corresponding metabolic capacities. Importantly, our model only considers qualitative conditions, i.e., the actual availability of metabolites, whereas the choice between alternative pathways can reflect adaptations to quantitative conditions, i.e., the concentration of metabolites [14],[47]. Considering this qualitative description of conditional-dependence of reactions, the association between the level of condition-dependent robustness and species' environmental diversity suggests that the former evolve as a result of a selection for alternating between nutritional sources. For most species examined the range of nutrients that can be consumed by a species can only partially explain the corresponding level of robustness (only about 20 percent of the reactions), where the complementary non condition-dependent robustness has arisen mainly to meet the metabolic requirements of a species. The association between the level of robustness and the corresponding metabolic capacity of a species can be explained either by a selection to increase flux – similarly to the effect of gene dosage [7] – or by selection to optimize the metabolic efficiency under given conditions, for example by alternating between routes in accordance with the corresponding substrate concentrations [47],[48]. Thus, the design of metabolic networks (as viewed by the presence of alternative pathways) represents a species-specific adaptation to both its needs and its environment.

Beyond the evolutionary implications of this study, additional applications can be withdrawn from the association between growth rate and topological properties (i.e., network robustness), as reported here. The observed association suggests that topological models can be used for predicting growth rate (for a broader spectrum of species than is currently possible with the existing range of stochiometeric models). An intriguing challenge is to develop supervised learning techniques to learn a predictive function for condition-dependent growth rate, given the topological characteristics of that environment-specific network and the species-specific growth potential under optimal conditions. Currently, there is a lack of systematic data of growth rate across media for a wide collection of species [54], needed for building the growth rate predictor suggested above. Hopefully, the future accumulation of such data will allow conducting such study.

## Methods

### Construction of species-specific metabolic networks and environments

Metabolic data on the enzymes and reactions in each species were collected from KEGG 24 (release 46) [55] for 487 bacterial organisms. The reaction scheme describing the substrates and products in each reaction was retrieved from the reaction_mapformula.lst file, describing only the main metabolites in each reaction (as in the KEGG pathway diagrams) and not the co-factors (e.g., H_2_O molecules). Metabolic networks were constructed as follows: Each enzyme is represented as a node in the network. Let E_1_ = e_11_, e_21_, … , e_n1_ denote the set of enzymes that catalyze reaction R_1_, and E_2_ = e_12_, e_22_, … , e_m2_ denote the set of enzymes that catalyze reaction R_2_. If a product of R_1_ is a substrate of R_2_, then edges are assigned between all nodes of E_1_ and all nodes of E_2_. Edges are also assigned within E_1_ nodes and within E_2_ nodes. A list of 86 target metabolites (Table S3) – that is, metabolites that are likely to be essential for growth in most species [56]–[58], was used for constructing species-specific target metabolite lists according to the intersection between the generic target metabolites and the metabolites that each species produces. Enzymes that are not relevant for the production of biomass metabolites were omitted from the networks. Such enzymes were identified by repeating the expansion of the network in a reverse manner, using the full networks and the set of biomass metabolites as seeds. Each reaction that participated in the production of biomass metabolites was added to the network. Thus, only reactions that had no part (direct or indirect) in the production of the biomass metabolites were omitted from the network. In the approach taken here, reactions that are not involved in the production of the biomass metabolites will have no effect on the network's viability following a deletion, hence would have no effect on our results. Using these effective reactions, we constructed the *effective network* further used throughout the analysis. For each network we computed modularity, centrality and connectivity. Modularity was computed using Newman's algorithm [59] as described in [60]. Centrality is computed by first determining all pairwise shortest paths using the Floyd–Warshall algorithm [61] and then calculating, for each node, its mean shortest path (MSP) distance to all other nodes in the network, denoting the node's centrality (within a specific network). In cases where the network has more than one connected component, nodes from two different components are assumed to have a distance of twice the maximal distance obtained within the components. The centrality of the network is the minimal MSP across all nodes.

Metabolic growth environments (rich media) were inferred for each species individually using the seed algorithm developed by [20], retrieving an ensemble of 487 species-specific rich growth media (one for each species). Other approaches for the construction of metabolic environments were additionally employed (Text S1 Note 2). To compute the species viability across all environments we tested the viability of each species over the set of 487 metabolic growth environments as follows: given a specific organism and an environment (set of external metabolites), an organism is considered viable in this environment if all its essential target metabolites are produced – this is examined by using a network expansion algorithm [15] that outputs an activated metabolic sub-network, and verifying that the expanded subnetwork produces all target metabolites. The *Environmental robustness* of each network is calculated as the corresponding fraction of viable environments.

### Computing topological network genetic robustness (NGR)

For each metabolic network (i.e., species), we compute *condition-dependent and independent NGR*. The species-specific NGR score is computed as follows: using its species-specific metabolic rich environment as the species' growth medium, each enzyme in the network examined is knocked out in turn and the expansion algorithm is used to evaluate if all target metabolites are produced in the perturbed network. If the whole list is still successfully produced, this enzyme is scored 1 (non-essential) and otherwise it is scored 0 (essential). The fraction of non-essential enzymes in the network is the network's *NGR* score. *Condition-independent NGR* is computed using the ensemble of all viable environments of a given species; i.e., we repeat the same knockout procedure in each viable environment. Subsequently, an enzyme is scored 1 if it is backed-up across all environments (non-essential) and 0 otherwise. The enzymes that are non-essential under all conditions are termed *condition-independent* and their fraction denotes the network's overall *condition-independent* NGR score. Enzymes are termed *condition-dependent* if they are found to be backed-up in some (but not all) of the viable media examined, per species, and their fraction in a given species is their *condition-dependent* NGR score. Notably, the species-specific rich media refers to the most optimal metabolic environment a species can have, i.e., an environment where all of its metabolic pathways have the potential to be active. Hence, reactions that are essential when the metabolic network works at full capacity will also be essential under less favorable conditions. The species-specific *condition-dependent and independent NGR* scores are listed in Table S1.

### Benchmarking the topology-driven predictions for reactions' essentiality against experimental data

Using the procedure described above we classify each reaction in each species as non-essential, essential or conditional-dependent (essential/non-essential). Non-essential and essential reactions (scoring either 1 or 0, respectively, i.e., non-essential or essential across all environments) of *E. coli* and *Bacillus subtilis* were assigned to the corresponding genes by parsing the KEGG ‘enzyme’ file (downloaded from ftp://ftp.genome.jp/pub/kegg/). The essentiality predictions for 327 (out of 530) *E. coli* reactions and 250 (out of 397) *Bacillus subtilis* reactions which are assigned to a single gene were compared with the pertaining experimental data. Experimental data from systematic gene knock-out studies of *E. coli* genes were retrieved from [62] and for the essentiality of *Bacillus subtilis* genes were retrieved from [63]. Accuracy was calculated as the fraction of true positives and true negatives out of all observations.

### Retrieving species-specific measures

Fractions of regulatory genes were taken from [36], describing the fraction of transcription factors out of the total number of genes in the organism as an indicator of transcriptional complexity (indirectly testifying to environmental variability) [37]. This is also the measure of transcriptional complexity. Environmental complexity values were obtained from [37], where the natural environments of 117 bacterial species were ranked based on the NCBI classification for bacterial lifestyle [64]. Growth rate: minimal duplication-time data, available for 109 species in the dataset were retrieved from [65]. Secondary metabolism: the fractions of enzymes involved in secondary metabolites were constructed by parsing KEGG data and counting for each species the number of enzymes which participate in pathways of secondary metabolism.

## Supporting Information

Text S1 Supplementary Notes, Tables and Figures.
(0.53 MB PDF)

Table S1 Genomic and ecological attributes of species in the analysis. This table displays the following attributes for the 487 bacterial species studied here: KEGG label, name, network size (number of reaction-nodes participating in the production of biomass metabolites), environmental robustness, condition-dependent NGR, condition-independent NGR (across 487 environments), condition-independent NGR (across 487+200000 environments), fraction of regulatory genes, environmental complexity score, lifestyle description, doubling time, oxygen requirements, centrality of the network, connectivity (mean rank) an modularity, correlation between reactions' essentiality and reactions' conservation, centrality and connectivity and the P-value for the correlation (spearman) . Values were computed and retrieved as described in the Methods section.
(0.09 MB TXT)

Table S2 Levels of evolutionary conservation (phylogenetic/phyletic distributions) and essentiality of reactions across species and environments.
(0.35 MB XLS)

Table S3 Full description and KEGG ID of the 86 target metabolites.
(0.01 MB PDF)

Table S4 List of all species which have an enzymes catalyzing reaction 1.14.16.1 (Phenylalanine 4-monooxygenase).
(0.03 MB PDF)

Table S5 List of human commensal and pathogens and the distribution and essentiality of Methenyltetrahydrofolate cyclohydrolase (EC 3.5.4.9) and Formyltetrahydrofolate synthetase (6.3.4.3) across these species.
(0.03 MB XLS)

Table S6 Distribution and essentiality of reactions across human commensal and pathogens organisms.
(0.06 MB XLS)
