# Supplementary material for: Decoupling Environment-Dependent and Independent Genetic Robustness across Bacterial Species
Source: PLoS Comput Biol. 2010 Feb 26;6(2):e1000690. doi: 10.1371/journal.pcbi.1000690 (PMC2829043; doi:10.1371/journal.pcbi.1000690)
Supplement: Text S1 — Supplementary Notes, Tables and Figures. (0.53 MB PDF) [file pcbi.1000690.s001.pdf]

Decoupling Environment-Dependant and Independent Genetic Robustness across Bacterial Species

S. Freilich*[1,2] , A. Kreimer*[3] , E. Borenstein[5,6], U. Gophna[4], R. Sharan[1] & E. Ruppin[1,2]

[1] The Blavatnik School of Computer Sciences, [2] School of Medicine, [3] School of Mathematical Science, [4] Department of Molecular Microbiology and Biotechnology, Faculty of Life Sciences, Ramat Aviv 69978, Israel. [5]Department of Biological Sciences, Stanford University, Stanford, CA 94305-5020 and [6]Santa Fe Institute, Santa Fe, NM 87501

*These authors contributed equally to this work

Supplementary notes.

Supplementary note 1: description of reactions' essentiality across species and environments

We examined for each species the essentiality of its reactions across all its viable environments (all files are available on http://www.cs.tau.ac.il/~jonatha6/publications/htmls_to_upload.zip).

The list of species/environments is presented in species_mapping.html, where each entry leads to the species-specific table describing the essentiality of each reaction (essential/non-essential reactions are denoted by red/green color respectively) over all viable environments. The list of reactions is presented in enzymes_mapping.html.

Supplementary note 2: Determining condition-essential reactions across viable environments

We recorded the accumulation of condition-essential/non-essential reactions across the viable environments of species. We observe that the number of condition-independent

non-essential reactions reaches a plateau, where beyond a certain number of environments the introduction of new conditions does not reveal additional condition-essential/non-essential reactions, i.e., reduce further the number of non-essential reactions. Supplementary Figure 1 (A, B) demonstrates this for *E. coli* and *B. thuringiensis*.

To further examine whether the range of 487 environments (as in main text) is indeed sufficient to reveal the large majority of condition-essential/non-essential reactions, we constructed a set of 20,000 random environments by shuffling the seeds from the original environments while maintaining the original metabolites' distribution overall seeds. That is, if a certain metabolite has X appearances over all 487 original environments, then it is randomly assigned to (X/487)*20000 environments. When computing the condition-independent NGR score following growth simulation in the 20000 growth media, we observe a strong correlation between the NGR score obtained while using the original environments and the NGR score obtained while using the random environments (0.58, *P* < 2e-16, Spearman; The correlation is even stringer when excluding 140 species for which we find no viable random environments: 0.79, *P* < 2e-16, Spearman). To examine whether using the random environment we find many additional condition-essential/non-essential reactions that are not found using the original environments we constructed a combined NGR score. The values obtained are very similar to these obtained across the 487 environments (Table S1), with a mean difference of 0.03. In *E. coli* and *B. thuringiensis* for example we compute NGR values of 0.787 and 0.759 across the 487 random environments and of 0.783 and 0.751 across the original and random environments (487+20,000) combined together (respectively). Supplementary Figure 1 (C, D) demonstrates that the saturation under the original 487 environments is almost identical when considering the additional 20,000 environments. This indicates that we cover, to a large extent, the potential nutrient-environments of each species and, consequently, are able to identify the large-majority of condition-essential/non-essential reactions.

For each species, NGR values computed over the random environments only (20,000) were compared to NGR values computed in the species-specific optimal environment, allowing to compute the fraction of condition essential/non-esential reactions (i.e., reactions essential in

at least one of the random environments and not essential in the optimal environment). We find a strong correlation between the fraction of condition-essential/non-essential and the environmental robustness calculated for the set of random environments (0.76, P < 2e-16, Spearman, Supplementary Note 2).

Supplementary note 3: Controlling for the effect of network size on the Centrality and connectivity

We observe a significant correlation between the NGR (fraction of non-essential reactions) and both the connectivity and centrality of the network (main text, Methods). Since both factors also exhibit correlation with network size (0.81 P < 2e-16 and 0.92 P < 2e-1 respectively, Spearman), we tested whether the original association remains significant following controlling for the effect of network size. We developed a linear regression model which computes the NGR as the function of (1) network-size; (2) network centrality; (3) networks' connectivity; (4) network's size and network's centrality; (5) network's size and network's connectivity. We applied a chi-square test to examine whether the combined models (4 and 5) provide better predictions than the individual models. Correlations values (Pearson) between observed and predicted condition-dependant NGR are: 0.66, 0.67, 0.78, 0.71, and 0.80 respectively. We find that the combined models (4 and 5), including topological information, significantly improves the accuracy of predictions then relying solely on network size (P values: 2e-16 and 3e-61, respectively; chi-square test), demonstrating that topological information is associated with NGR beyond the mutual association with network size

Supplementary note 4: Relationship between condition-essentiality and topological properties of reactions

Distribution of the species-specific correlation values (Spearman) between condition-essentiality (fraction of non-essential appearances across all species' viable environments) and centrality and connectivity was recorded across all species. Connectivity: We observe a significant positive correlation between condition-essentiality of a reaction and node's rank in 429/480 species (90%), mean correlation: 0.2; maximal correlation: 0.4. Centrality: We observe a negative correlation between

condition-essentiality of a reaction and the mean shortest path between the reaction and all pairwise combinations in the network in 466/480 species (97%), the negative correlation is significant in 367 species (76%); mean correlation: -0.17; maximal correlation: -0.4. Species-specific correlation values are shown in Table S1.

Supplementary note 5: Enrichment of essential/non-essential reactions across KEGG-pathways

Reactions were classified into KEGG pathway-types categories (http://www.kegg.jp/kegg/pathway.html#metabolism), where only pathway-types involved in the production of biomass metabolites are considered. The distribution of essential/non-essential reaction across the categories is shown in Supplementary Figure 2. Enrichments of super-pathways in non-essential versus essential reactions (conditional and non-conditional), compared with a random distribution of the contributing reactions across categories, were computed using Fisher's exact test for testing the null of independence of rows and columns in a contingency table with fixed marginal. Distribution values and P values from Fisher's exact test are shown in Table 1.

Supplementary note 6: Distribution of non-essential reactions across all reactions, oxygen-utilizing reactions, and ATP-utilizing reactions

Identification of reactions was done according to KEGG 'enzyme' file where oxygen/ATP/NAD/NADP-utilizing reactions are reactions that use oxygen/ATP as a substrate, respectively. NAD--utilizing reactions are reactions which use either NAD or NADH as a substrate. NADP--utilizing reactions are reactions which use either NADP or NADPH as a substrate. The fractions of non-essential reactions in each category are listed in Table 3. Significance differences at the mean number of non-essential appearances are observed between all reactions and oxygen-utilizing reactions (Wilcoxon test: 5e-5); between all reactions and ATP-utilizing reactions (Wilcoxon test: 1.2e-4); between all reactions and NAD-utilizing reactions (Wilcoxon test: 5e-3); No significant difference is observed between all reactions and NADP-utilizing reactions. For each reaction class we also recorded the mean number of non-essential reactions at aerobic, anaerobic, and

facultative bacteria. We do not observe significant differences between the different categories of respiration mode (oxygen/ATP/NAD/NADP-utilizing reactions).

Supplementary note 7: Phenylalanine 4-monooxygenase as an example of non-essential reaction across aerobic bacteria

Phenylalanine 4-monooxygenase (EC 1.14.16.1) catalyses the following reaction of converting phenylalanine into tyrosine:

L-phenylalanine + tetrahydrobiopterin + O2 = L-tyrosine + 4a-hydroxytetrahydrobiopterin

This reaction, limited to respiratory-bacteria, is non-essential across the large majority of species where it is found (82/98), where all 82 species maintain an alternative conserved route for tyrosine production from prephenate in a reaction catalyzed by prephenate dehydrogenase (1.3.1.12). None of the 36 anaerobic bacteria which contain prephenate dehydrogenase have alternative routes for this reaction, which is essential across all anaerobes in our data. The distribution of reactions and their essentiality across species is described at Table S4.

Supplementary note 8: Controlling for the effect of network size on the association between environmental diversity and the fraction of condition-essential/non-essential reactions.

We observe a significant correlation between the fraction of condition-essential/non-essential reactions and environmental diversity (0.81, $P < 2e-16$, Spearman). Since both factors also exhibit correlation with network size (fraction of condition-essential/non-essential reactions exhibits a correlation of 0.51 , $P < 2e-16$, and environmental diversity a correlation of 0.58 , $P < 2e-16$, Spearman correlation), we tested whether the original association remains significant following controlling for the effect of network size. Indeed, a linear regression model that computes the fraction of condition-essential/non-essential genes as the function of both environmental diversity and network size does not improve the prediction accuracy obtaining using environmental robustness solely (chi-square test).

Supplementary note 9: Estimating the mutual associations between growth-rate and the fractions of secondary-metabolites, and condition-independent NGR.

We developed a linear regression model which computes the condition-independent NGR as the function of (i) log of minimal doubling time (growth rate); (ii) fraction of reactions involved in the synthesis of secondary metabolites; and (iii) both parameters. We applied a chi-square test to examine whether the combined model (iii) provides better prediction than the individual model. We find that model (iii) significantly improves the accuracy of predictions of model (i) and (ii) (P values: 1e-5 and 6.9e-12, respectively), demonstrating that the combination of both factors significantly improve the model.

Supplementary note 10: Estimating the mutual associations between growth-rate, the fractions of secondary-metabolites, environmental robustness and condition-dependent NGR.

We developed a linear regression model which computes the condition-dependant NGR as the function of (i) log of minimal doubling time (growth rate); (ii) fraction of reactions involved in the synthesis of secondary metabolites; and (iii) both parameters. We applied a chi-square test to examine whether the combined model (iii) provides better prediction than the individual model. Correlations values (Pearson) between observed and predicted condition-dependant NGR are: 0.23, 0.07, and 0. 24 respectively. We find that model (iii) does not significantly improves the accuracy of predictions then relying solely on growth rate, demonstrating that the combination of both factors does not significantly improves the model.

Yet, we observe a significant correlation (P = 0.01) of 0.23 between growth rate and condition-dependant NGR. This correlation can be explained in terms of the correlation between growth rate (log minimal doubling time) and environmental robustness (-0.31 P value: 1e-3, Pearson). We developed a linear regression model which computes the condition-dependant NGR as the function of (i) log of doubling time; (ii) environmental robustness; and (iii) both parameters. We applied a chi-square test to examine whether the combined model (iii) provides better prediction than the individual model. Correlations values (Pearson) between observed and predicted condition-dependant NGR are: 0.23, 0.52, and 0, 53 respectively. We find that model (iii) does not significantly

improves the accuracy of predictions then relying solely on environmental robustness, demonstrating that the combination of both factors does not significantly improves the model.

Supplementary note 11: Computing the number of gene-copies in essential versus non-essential reactions

To study adaptive versus intrinsic selection for robustness we examined the association between pathway-level robustness (the existence of alternative pathways, NGR) and gene-level robustness (the existence of duplicate genes or of functional-analogs). The assignment of genes into reactions was extracted from the species-specific gene files (ftp://ftp.genome.jp/pub/kegg/genes/organisms/; *.ent files). Reactions that describe multi-subunit complexes (where multi gene-copy does not imply on gene-level buffering) were excluded from the analysis. We find that the fraction of multi-gene copies of essential reactions is not significantly different than the fraction of multi-gene copies of non-essential reactions (Fisher exact test).

Supplementary note 12: NGR of species exposed to high-levels of radiation

The immediate gain of reaction's dispensability is robustness in face of genetic perturbations. Despite the prevalence of robustness in metabolic-networks across species, there is no evidence that this phenotype is the outcome of direct selection in favor of reduced susceptibility to mutations. To test whether we can find such evidence, we quantified the level of NGR at *Rubrobacter xylanophilus* and *Deinococcus radiodurans* - extermophyls which are exposed to high levels of radiation [1]. Perhaps surprisingly, the NGR values of both species do not significantly differ from those observed in other bacteria. (0.74 and 0.77 respectively, compared with a mean value of 0.75 over all species). In the absence of growth rate data for these organisms, we could not examine how well does their NGR score agree with their metabolic requirements. The distribution of multi-gene copies of essential and non-essential reactions in these species also does not provide any evidence for a preferential distribution that would testify to adaptive evolution of robustness in these species.

Supplementary note 13: Identifying reactions which are essential in human pathogen

We listed all human commensals and pathogens (8 and 86 respectively) from our database. The full list is available at Table S5. In the table we provide the species-specific essentiality scores of Methenyltetrahydrofolate cyclohydrolase (EC 3.5.4.9) and Formyltetrahydrofolate synthetase (6.3.4.3).

For each reaction in the data we calculated in how many commensals and in how many pathogens it appears and its mean essentiality score across species and environments. We calculated for each reaction the ratio between mean essentiality in commensals to mean essentiality in pathogens; high ratio scores denote reactions which are generally non-essential in commensals and essential in pathogens. These reactions can thus be suspected as drug targets. The ratio between essentiality score is provided at Table S6.

Tables

KEGG pathway-types	Fraction of non-essential reactions	Fraction of essential reactions	Enrichment (P values)	
			Non-essential	Essential
Lipid_metabolism	0.773922	0.226078	0.02	0.98
Amino_acid_metabolism	0.762833	0.237166	0.95	0.05
Amino acid biosynthsis	0.404854	0.595145	1	0
Amino acid degradation	0.872826	0.127173	3.4E-80	1
Glycan_biosynthesis_metabolism	0.641232	0.358768	1	2.26E-90
Energy_metabolism	0.856736	0.143264	1.8E-203	1
Metabolism_of_cofactors_and_vitamins	0.722688	0.277312	1	1.04E-53
Nucleotude_netabolism	0.927536	0.072463	0	1
Carbohydrate_metabolism	0.898662	0.101337	0	1
Metabolism_of_other_amino_acids	0.808850	0.191149	1.02E-30	1

Table 1. Distribution of non-essential and essential reactions across KEGG pathway categories. Condition-essential reactions are considered essential. Enrichment of non-essential/essential reactions in each category was tested using Fisher's exact test.

Lifestyle categories (NCBI)	# of species with significant correlation	# of species with non-significant correlation	Enrichment (P values)	
			Correlated -species	Non-correlated species
Aquatic	60	11	0.717532	0.409728
Host-associated	138	54	3.69E-07	1
Multiple	139	14	0.999755	0.000692
Specialized	21	3	0.815669	0.381135
Terrestrial	32	0	1	0.001871
unknown	13	2	0.763432	0.505857

Table 2. Distribution of significant and non-significant spearman correlations for the relationships between evolutionary conservation (phyleogentic-distribution) and species-specific condition essentiality across lifestyle categories. Enrichment was tested using Fisher's exact test.

	All species (487)	Aerobic bacteria (154)	Facultative bacteria (196)	Anaerobic bacteria (55)
All reactions (2283)	0.877282	0.868792	0.873077	0.836493
Oxygen-utilizing reactions (267)	0.96185	0.948501	0.961593	0.903045
ATP-utilizing reactions (255)	0.788592	0.770434	0.782588	0.758509
NAD-utilizing reactions (280)	0.927753	0.926668	0.922247	0.8643296
NADP-utilizing reactions (261)	0.863549	0.848456	0.860835	0.819639

Table 3. Mean fraction of non-essential reactions over reactions utilizing specific substrates.

Figures

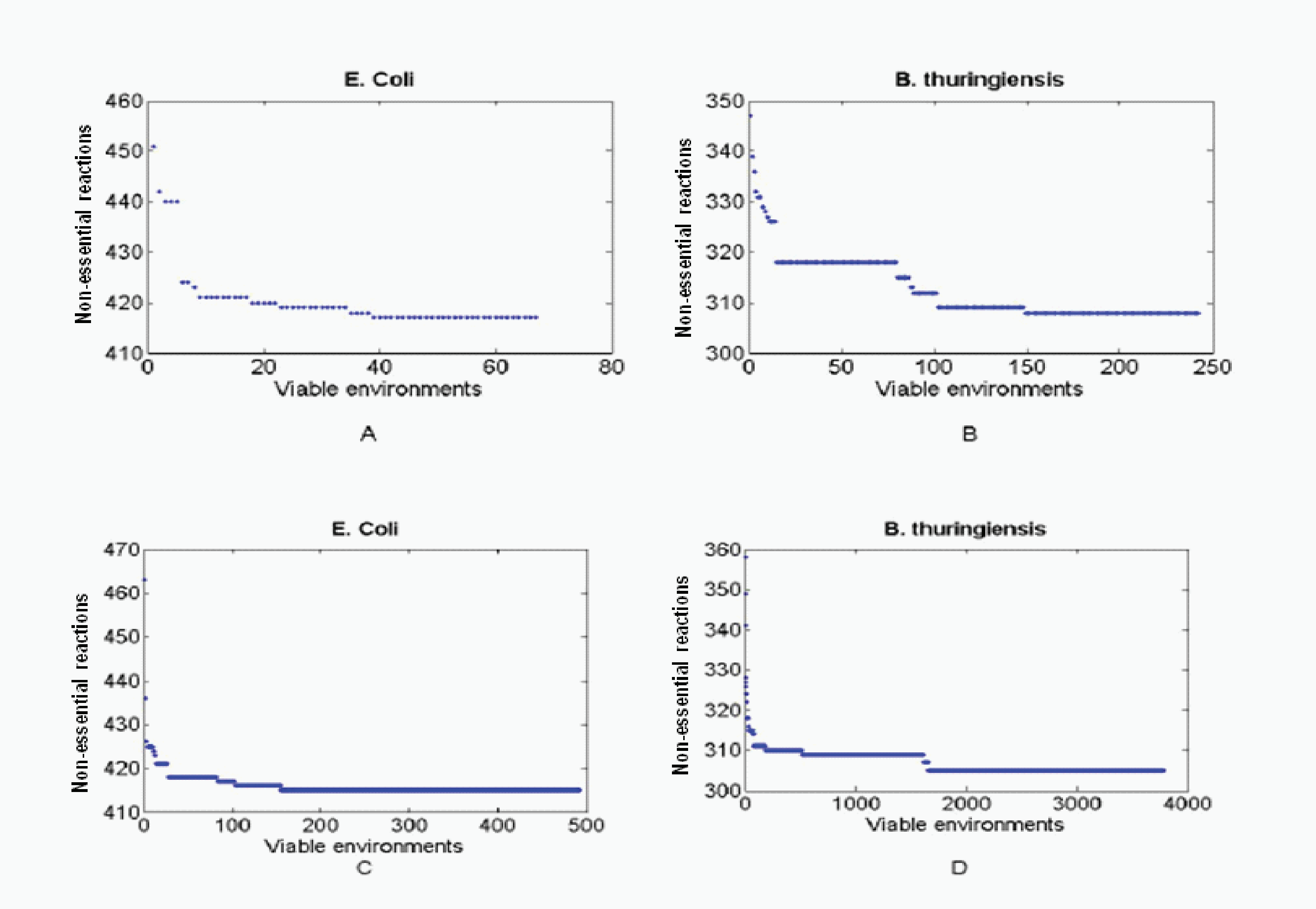

Figure 1. Cumulative fraction of non-essential reactions across all viable environments of organisms. A, B – considering the original 487 environments; C, D – considering the original 487 environments together with 20000 random environments. We record 67 and 493 viable environments for *E. coli* and 242 and 3783 viable environments for *B. thuringiensis* across the original and random environments, respectively.

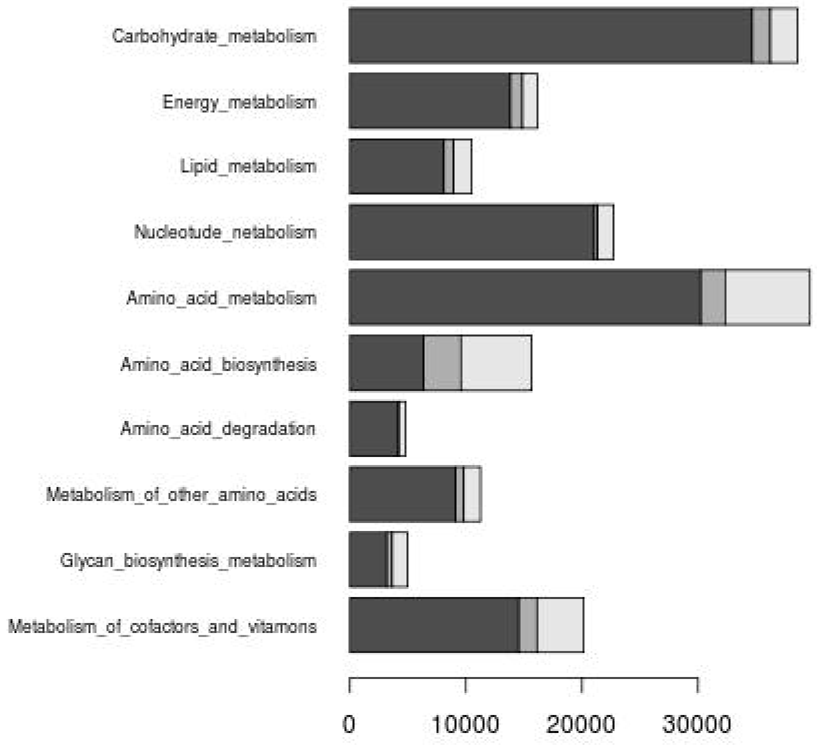

Figure 2. Metabolic-network robustness in KEGG pathway-types categories, across species and growth environments, showing for each categories the fractions of non-essential reactions (black), condition-essential/non-essential reactions (dark grey), and essential reactions (light grey).

A

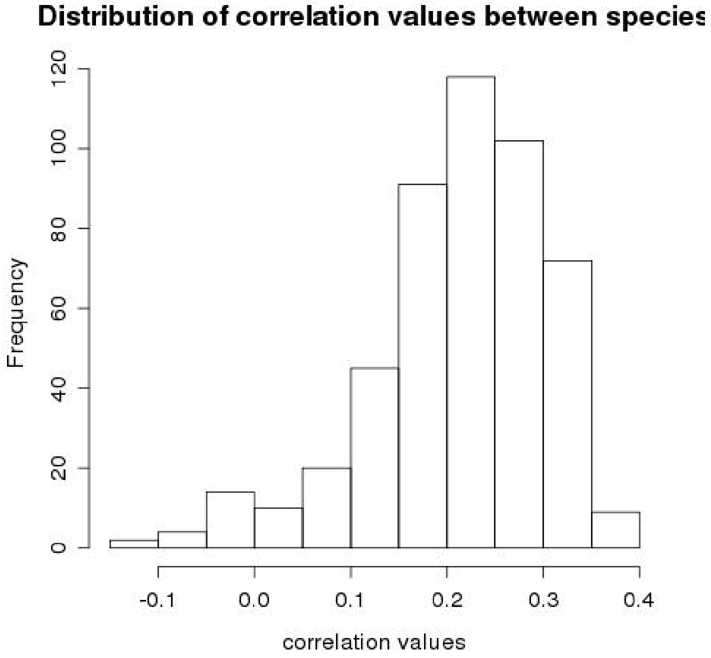

B

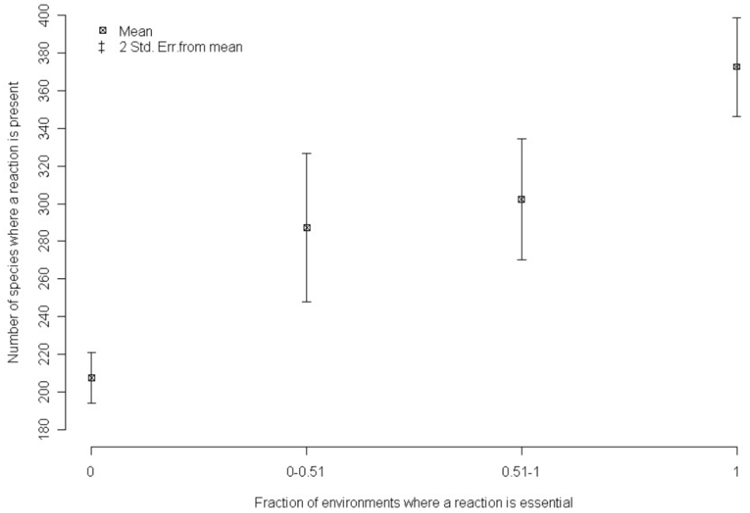

Supplementary Figure 3. Relationship between condition-essentiality and evolutionary conservation of reactions. (A) Distribution of the species-specific correlation values (spearman; essentiality across environments per species versus evolutionary conservation of reactions). We observe only 84 species where the correlation is not significant

(P>0.05). Species-specific correlation values are shown in Table S1; The enrichment of lifestyle classes in the 84 species devoid of these correlations is shown in Table 2. (B) The relationship between condition-essentiality and evolutionary conservation in *E. coli*. Reactions that are essential across many tissues are more widely conserved.

References

1. Ferreira, A.C., et al., *Characterization and radiation resistance of new isolates of Rubrobacter radiotolerans and Rubrobacter xylanophilus.* Extremophiles, 1999. **3**(4): p. 235-8.
