# Supplementary material for: Decoupling Environment-Dependent and Independent Genetic Robustness across Bacterial Species
Source: PLoS Comput Biol. 2010 Feb 26;6(2):e1000690. doi: 10.1371/journal.pcbi.1000690 (PMC2829043; doi:10.1371/journal.pcbi.1000690)
Supplement: Table S3 — Full description and KEGG ID of the 86 target metabolites. (0.01 MB PDF) [file pcbi.1000690.s004.pdf]

KEGG #	Description
C00037	Glycine
C00041	L-Alanine
C00183	L-Valine
C00123	L-Leucine
C00407	L-Isoleucine
C00065	L-Serine
C00188	L-Threonine
C00079	L-Phenylalanine
C00082	L-Tyrosine
C00078	L-Tryptophan
C00097	L-Cysteine
C00073	L-Methionine
C00047	L-Lysine
C00062	L-Arginine
C00135	L-Histidine
C00049	L-Aspartate
C00025	L-Glutamate
C00152	L-Asparagine
C00064	L-Glutamine
C00148	L-Proline
C00020	AMP
C00360	dAMP
C00131	dATP
C00002	ATP
C00144	GMP
C00362	dGMP
C00286	dGTP
C00044	GTP
C00239	dCMP
C00055	CMP
C00458	dCTP
C00063	CTP
C00364	dTMP
C00459	dTTP
C00105	UMP
C00075	UTP
C06040	Diglucosyl-diacylglycerol
C00116	Glycerol
C05980	Cardiolipin
C00350	Phosphatidylethanolamine
C00641	1,2-Diacyl-sn-glycerol
C16221	(2E)-Octadecenoyl-[acp]
C00249	Hexadecanoic acid
C05764	Hexadecanoyl-[acp]
C05890	Undecaprenyl-diphospho-N-acetylmuramoyl-(N-acetylglucosamine)-L-
C05894	Undecaprenyl-diphospho-N-acetylmuramoyl-(N-acetylglucosamine)-L-
C05899	Undecaprenyl-diphospho-N-acetylmuramoyl-(N-acetylglucosamine)-L-
C00234	10-Formyltetrahydrofolate
C00003	NAD+
C00004	NADH

C00006	NADP+
C00005	NADPH
C00008	ADP
C00035	GDP
C00112	CDP
C00015	UDP
C00043	UDP-N-acetyl-D-glucosamine
C00748	Siroheme
C01050	UDP-N-acetylmuramate
C00054	Adenosine 3',5'-bisphosphate
C00024	Acetyl-CoA
C00016	FAD
C15672	Heme O
C00399	Ubiquinone
C00255	Riboflavin
C02839	L-Tyrosyl-tRNA(Tyr)
C00886	L-Alanyl-tRNA
C02163	L-Arginyl-tRNA(Arg)
C03402	L-Asparaginyl-tRNA(Asn)
C03125	L-Cysteinyl-tRNA(Cys)
C02282	Glutaminyl-tRNA
C02412	Glycyl-tRNA(Gly)
C03127	L-Isoleucyl-tRNA(Ile)
C02047	L-Leucyl-tRNA
C01931	L-Lysyl-tRNA
C02430	L-Methionyl-tRNA
C03511	L-Phenylalanyl-tRNA(Phe)
C02702	L-Prolyl-tRNA(Pro)
C02553	L-Seryl-tRNA(Ser)
C02992	L-Threonyl-tRNA(Thr)
C03512	L-Tryptophanyl-tRNA(Trp)
C02554	L-Valyl-tRNA(Val)
C02984	L-Aspartyl-tRNA(Asp)
C02987	L-Glutamyl-tRNA(Glu)
C00039	DNA
C00046	RNA
