# Supplementary material for: Decoupling Environment-Dependent and Independent Genetic Robustness across Bacterial Species
Source: PLoS Comput Biol. 2010 Feb 26;6(2):e1000690. doi: 10.1371/journal.pcbi.1000690 (PMC2829043; doi:10.1371/journal.pcbi.1000690)
Supplement: Table S4 — List of all species which have an enzymes catalyzing reaction 1.14.16.1 (Phenylalanine 4-monooxygenase). (0.03 MB PDF) [file pcbi.1000690.s005.pdf]

species	Respiratory	Essentiality	Essentiality of reaction 1.3.1.12
aav	Aerobic	1	1
aba	unknown	1	1
ade	Facultative	1	1
aha	Facultative	1	1
ajs	Aerobic	0	0
baa	Facultative	1	1
bam	Facultative	1	1
ban	Facultative	1	1
bar	Facultative	1	1
bat	Facultative	1	1
bba	Aerobic	0	0
bca	Aerobic	1	1
bce	Aerobic	1	1
bch	unknown	1	1
bcn	unknown	1	1
bcz	Aerobic	1	1
bma	unknown	1	1
bml	unknown	1	1
bmn	unknown	1	1
bmv	unknown	1	1
bpd	Aerobic	1	1
bpl	Aerobic	1	1
bpm	Aerobic	1	1
bps	Aerobic	1	1
bte	Aerobic	1	1
btk	Facultative	1	1
btl	Facultative	1	1
bur	Facultative	1	1
bvi	Facultative	1	1
bxe	Aerobic	1	1
ccr	Aerobic	1	1
chu	Aerobic	1	1
cps	Facultative	1	1
cvi	Facultative	1	1
eli	Aerobic	1	1
fps	Aerobic	0	0
gfo	Aerobic	1	1
hch	Facultative	0	0
hne	Aerobic	1	1
ilo	Aerobic	1	1
mlo	Aerobic	1	1
mmr	Facultative	1	1
mxa	Aerobic	1	1
nar	Aerobic	1	1
nfa	Aerobic	1	1
pae	Aerobic	1	1

pat	Aerobic	1	1
pau	Aerobic	0.285714	0
pcr	unknown	0.5	1
pen	unknown	1	1
pfl	Aerobic	1	1
pfo	Aerobic	1	1
pha	Aerobic	1	1
pmy	Aerobic	0	0
pna	Aerobic	0	0
pol	Aerobic	1	1
ppf	Aerobic	0	0
ppr	Facultative	1	1
ppu	Aerobic	1	1
prw	Aerobic	0	0
psb	Aerobic	1	1
psp	Aerobic	1	1
pst	Aerobic	1	1
reh	Facultative	1	1
ret	Aerobic	1	1
reu	Facultative	1	1
rle	Aerobic	1	1
rme	Facultative	1	1
rso	Aerobic	1	1
sal	Aerobic	1	1
saz	Facultative	1	1
sbl	Facultative	1	1
sbm	Facultative	0.5	0
scl	Aerobic	0.5	0
sdn	Facultative	1	1
sfr	Facultative	1	1
she	Facultative	1	1
shm	Facultative	1	1
shn	Facultative	1	1
shw	Facultative	1	1
slo	Facultative	1	1
son	Facultative	1	1
spc	Facultative	1	1
spl	Facultative	0	0
sru	Aerobic	1	1
sse	Facultative	0	0
vch	Facultative	1	1
vco	Facultative	0	0
vei	unknown	0.25	1
vpa	Facultative	1	1
vvu	Facultative	1	1
vvy	Facultative	1	1
xac	Aerobic	1	1

xcb	Aerobic	1	1
xcc	Aerobic	1	1
xcv	Aerobic	1	1
xom	Aerobic	1	1
xoo	Aerobic	1	1
